# Therapy of spinal cord injury by folic acid polyethylene glycol amine-modified zeolitic imidazole framework-8 nanoparticles targeted activated M/Ms

**DOI:** 10.3389/fbioe.2022.959324

**Published:** 2022-09-15

**Authors:** Qi Li, Yue Guo, Chang Xu, Jiachen Sun, Fanzhuo Zeng, Sen Lin, Yajiang Yuan

**Affiliations:** ^1^ Department of Orthopedics, First Affiliated Hospital of Jinzhou Medical University, Jinzhou, China; ^2^ Key Laboratory of Medical Tissue Engineering, Jinzhou Medical University, Jinzhou, China

**Keywords:** spinal cord injury, microglia/macrophages, metal-organic frameworks, inflammation, apoptosis

## Abstract

Excessively activated microglia/macrophages (M/Ms) re-establish the proinflammatory microenvironment that exacerbates motor and/or sensory dysfunction after spinal cord injury (SCI). Thus, proinflammatory M/Ms-suppressed treatments may be effective strategies for SCI. However, the utilization of anti-inflammatory drugs for clinical approaches and biomedical research has side effects, such as nephrotoxicity and hepatotoxicity. In this study, we fabricated folic acid-polyethylene glycol (FA-PEG) amine-modified zeolitic imidazole framework-8 (ZIF-8) nanoparticles (FA-PEG/ZIF-8) and found that it effectively restored function *in vivo*. FA-PEG/ZIF-8 treatment significantly eliminated proinflammatory M/Ms without targeting other nerve cells and downregulated inflammation in the injured lesion. Furthermore, FA-PEG/ZIF-8 caused little toxicity in SCI mice compared to normal mice. These results suggest that FA-PEG/ZIF-8 has the potential to help recover from early-stage SCI by suppressing proinflammatory M/Ms.

## Introduction

Microglia/macrophages (M/Ms) play a key role in the pathogenesis of many inflammation-related diseases, including spinal cord injury (SCI) (David and Kroner, 2011; Plemel et al., 2014; Fan et al., 2018). The mechanisms of SCI are complex and not fully understood (Dietz and Fouad, 2014; Ahuja et al., 2017), but a well-characterized initiating factor is M/Ms in the injured spinal cord (McDonald and Sadowsky, 2002). Under the predominant state of proinflammatory M/Ms, the microenvironment of the injured spinal cord is detrimental to axon regeneration and tissue repair (David and Kroner, 2011; Plemel et al., 2014; Fan et al., 2018). The phenotype of M/Ms, as proinflammatory and anti-inflammatory polarization, has a different capability to exert functions in SCI. Proinflammatory M/Ms, their induction of nitric oxide synthase (iNOS), and interleukin-1β (IL-1β) have aggravating effects on SCI. The highly expressed arginase-1 (Arg-1), interleukin-10 (IL-10), and anti-inflammatory M/Ms can promote regeneration and inhibit inflammation after SCI. Proinflammatory cytokines secreted from proinflammatory M/Ms are forcefully increased within a few minutes to 1 day in the injured spinal cord following SCI (Tang and Le, 2016), leading to motor dysfunction (Walker and Lue, 2015). Therefore, promoting recovery from injury-induced proinflammatory M/Ms has become a research hotspot for developing new methods to mitigate SCI damage.

Nanomaterials, as a new type of artificial treatment, have the advantages of low cost, high stability, excellent durability, and multifunctionality compared with natural drugs (Wu et al., 2019). Therefore, the invention of nanomaterials has recently shown great promise in the field of regenerative medicine. Metal–organic frameworks (MOFs) are considered a possible transmission (Blight et al., 2013), and zeolitic imidazolate framework-8 (ZIF-8) has shown excellent performance in drug delivery (Abdelhamid, 2021; Huan et al., 2021; Ke et al., 2021). It has been reported that modified ZIF-8 can inhibit tumor proliferation and increase the survival of mice (Zheng et al., 2016). However, the use of ZIF-8 to selectively target a certain kind of cell is challenging due to the limitations of nanomaterials. Folate receptors (FRs) are widely expressed on the membrane of numerous immune cells, and mediate selective phagocytosis into cells, especially in M/Ms (Hansen et al., 2015; Mohammadi et al., 2016; Chandrupatla et al., 2019; Chen et al., 2020). Folic acid-polyethylene glycol (FA-PEG) is recognized as a stable and compatible solution. FA-PEG modified the surface of ZIF-8 (FA-PEG/ZIF-8), which may be a novel nanomaterial platform for the targeted treatment of M/Ms during SCI recovery.

In the present study, we conceptually created artificial nanomaterials based on a *de novo* design strategy: We synthesized *in situ* FA-PEG-modified ZIF-8 nanoparticles (FA-PEG/ZIF-8) with M/Ms-targeting ability. These nanoparticles have significant therapeutic effects *in vivo* and *in vitro* without any apparent toxicity. This study provides an attractive strategy for developing nanoparticle-based nanomaterial systems, which can serve as a blueprint for next-generation nanomedicines for the treatment and prevention of M/Ms-related diseases.

## Materials and methods

### Chemical reagents

Zn(NO_3_)_2_·6H_2_O, dimethyl sulfoxide (DMSO), methanol, phosphate-buffered saline (PBS), 3-(4, 5-dimethylthiazol-2-yl)-2, 5-diphenyltetrazolium bromide (MTT), ECL detection kit, and lipopolysaccharide (LPS) were purchased from Solarbio Biochemical Co., Ltd. (China). Fetal bovine serum (FBS), trypsin/EDTA, Dulbecco’s modified Eagle’s medium (DMEM), and penicillin/streptomycin were purchased from Sigma-Aldrich Biotech., Ltd. (USA). A BCA protein assay kit was purchased from Beyotime Biotech (China). FA-PEG (MW = 2,000 Da) was purchased from Fan Shuo Biotech., Shanghai (China). Anti-CD11b antibody (NO. ab184308), anti-Iba-1 antibody (NO. ab178846), anti-Arg-1 antibody (NO. ab239731), anti-iNOS antibody (NO. ab210823), anti-NeuN antibody (NO. ab177487), anti-GFAP antibody (NO. ab7260), anti-MBP antibody (NO. ab218011), and anti-IL-1β antibody (NO. ab254360) were obtained from Abcam (USA). The anti-cleaved-caspase-3 antibody (NO. 9661S) was obtained from Cell Signaling Technology (UK).

### Synthesis and characterization of FA-PEG/ZIF-8

Zn(NO_3_)_2_·6H_2_O (0.66 mM) and 2-methylimidazole (24.36 mM) were, respectively, dissolved in 0.8 ml methanol and slowly stirred for 10 min. The deposition (ZIF-8) was filtrated from the solution, rewashed with methanol and centrifuged 3 times, and dried at 60°C under vacuum. Followed by adding to FA-PEG solution, the mixture was sonicated and slowly stirred for 2 days. The product was rewashed with deionized water, centrifuged 3 times, and then dried at 40°C under vacuum. The morphology and particle-size distribution were tested using scanning electron microscope (SEM) and transmission electron microscope (TEM), and dynamic light scattering (DLS), respectively. Fourier-transform infrared spectrophotometer (FTIR) spectra were used to determine the presence of FA-PEG in the ZIF-8 matrix. X-ray diffraction (XRD) was used to determine the crystal structure of FA-PEG in the ZIF-8 matrix.

### Culture of RAW264.7 cells

RAW264.7 macrophage cells were incubated in 96-well plates in the medium (Dulbecco’s modified Eagle’s medium (DMEM), 10% fetal bovine serum (FBS), and 200 μL penicillin/streptomycin) for 12 h. Then, the medium was cultured with LPS (1 μg/ml) for 1 day, following by various concentrations of FA-PEG (1, 10, 50, 100, 500, and 1,000 μg/ml), ZIF-8 (1, 5, 10, 20, 30, and 50 μg/ml), and FA-PEG/ZIF-8 (0.1, 0.5, 1, 2, 5, 10, and 20 μg/ml) and incubated in 5% CO_2_ at 37°C for 1 day. The next day, we added 150 μL DMSO for detecting cell viability followed by adding 3-(4,5)-dimethylthiahiazo (-z-y1)-3,5-di-phenytetrazoliumromide (MTT) solution to the medium for 6 h.

### Annexin V phosphatidylserine apoptosis assay

RAW264.7 cells were cultured in plates with the medium for 12 h. Next, the cells were stimulated with LPS without or with FA-PEG/ZIF-8 for 24 h. Briefly, after 24 h of treatment, the samples were washed three times with PBS and stained using an Annexin V-FITC apoptosis detection kit (Beyotime, China) according to the manufacturer’s protocol. Annexin V-FITC and PI-labeled cells were identified using flow cytometry (BD FACSVerse; BD Biosciences, USA), and the data were processed and analyzed using FlowJo software.

### Animal treatments

Male C57BL/6 mice (6–8 weeks old, 22–30 g) were fed in a controlled place with standard rodents. The animals are kept at 22 ± 1°C, 12 h light and 12 h dark cycle. A 2-mm diameter and 10 g impounder were filled on the T9-T10 spinal cord from 25 mm height, resulting in spinal cord moderate contusion. The bladder was massaged twice a day until bladder function was restored to normal. The SCI group was intravenously injected with physiological saline daily. The FA-PEG/ZIF-8 group was intravenously injected with different concentrations of these nanoparticles.

### Behavioral assessment

The mice’s behavior was determined by the BASSO MOUSE SCALE (BMS). The double-blind assessment was used at 0, 1, 3, 7, 14, 21, and 28 days after injury. The 0 point shows that the mice had no ankle movement, and the 9 point demonstrates normal function.

At 28 days after injury, footprint analysis was performed. The mice were coated with different dyes on their front and rear limbs, placed on absorbent paper surrounded by wooden boards, and requested to walk in a straight line (front limbs were coated with black dye and rear limbs were coated with red dye).

### Histological staining and immunofluorescence staining

The mice were anesthetized by urethane (20%, 5 ml/kg) 7 days after injury. 5-mm segments of the spinal cord including the injury lesion were taken. The segments were soaked in 4% paraformaldehyde for 3 days and added to 30% sucrose in 4% paraformaldehyde for 3 days. For hematoxylin and eosin (H&E) staining, 5-μm frozen sections were dried at room temperature for 30 min and immersed in hematoxylin for 6 min, and the slides were sluiced in running water for 10 s. The sections were differentiated in HCl/95% alcohol (1:50) solution for 5 s. After washing in running water for 25 min, the slides were restained with eosin and then fixed with neutral balsam after dehydration *via* 75% alcohol, 95% alcohol, and 100% alcohol and transparency with xylene. For immunofluorescent analysis, 5 μm sections were blocked with 5% normal goat serum for 1 h and incubated overnight at 4°C with primary antibodies. The next day, the tissues were rewashed with PBS and incubated with Alexa Fluor-488 or Alexa Fluor-568 at room temperature for 2 h. The nucleus was dyed with DAPI solution (1:1,000). The calculation result was found using the positive cell rate per 0.05 mm^2^. The absolute cell number counts and densities were calculated using the optical fractionator component of ImageJ2x software. The sampling scheme chosen ensured that the sample concentration remained constant for each section. The relative optical density was analyzed by ImageJ2x software (National Institute of Health, Bethesda, MD, USA).

### Quantitative real-time PCR

The tissue was taken 7 days after injury in this experiment for PCR. The relative expressions of the genes were normalized to the gene named ribosomal protein S18 (RPS18), and the target genes were compared with the corresponding target genes from the group using the (1 + e)^−ΔΔCT^ formula. The following oligonucleotide primers were listed in Table 1.

**TABLE 1:** Primer sequences used for quantitative real-time PCR.

Gene	Forward primer (5′ to 3′)	Reverse primer (5′ to 3′)
IL-1β	CCT​GTG​CTG​TCG​GAC​CCA​TA	CAG​GCT​TGT​GCT​CTG​CTT​GTG​A
iNOS	TTT​GCC​AAT​TCA​TTA​CTT​CCA	ATC​ACA​CCG​CCT​CCT​GAT​TCC
Arg-1	CTC​CAA​GCC​AAA​GTC​CTT​AGA​G	AGG​AGC​TGT​CAT​TAG​GGA​CAT​C
IL-10	CTA​TGC​TGC​CTG​CTC​TTA​CTG​AC	CGG​AGA​GAG​GTA​CAA​ACG​AGG
RPS18	GCA​ATT​ATT​CCC​CAT​GAA​G	GGC​CTC​ACT​AAA​CCA​TCC​AA

### *In vivo* biocompatibility evaluation

To evaluate the biocompatibility of FA-PEG/ZIF-8 *in vivo*, the mice were intravenously administered with FA-PEG/ZIF-8 at a single dose of 100 μg/kg. The mice injected with PBS were considered the normal group. One month post injection, the blood samples were collected for complete blood panel analysis and serum biochemistry test. The serum biochemistry test included two important indicators of hepatic function aspartate aminotransferase (AST) and alanine aminotransferase (ALT) and two indicators of kidney function as blood urea nitrogen (BUN) and creatinine (CRE).

### Statistical analysis

Data were expressed as mean±SD and analyzed by SPSS 21.0. Student’s t-test and one-way ANOVA tested the data of two groups and more groups. Moreover, the BMS scores and RT-qPCR analysis were analyzed by the two-way ANOVA (repeated measures) with Bonferroni’s post-tests and Wilcoxon’s rank-sum test, respectively. *P* < 0.05 was considered statistically significant.

## Results and discussion

### Synthesis and characterization of the materials

**FIGURE 1:**
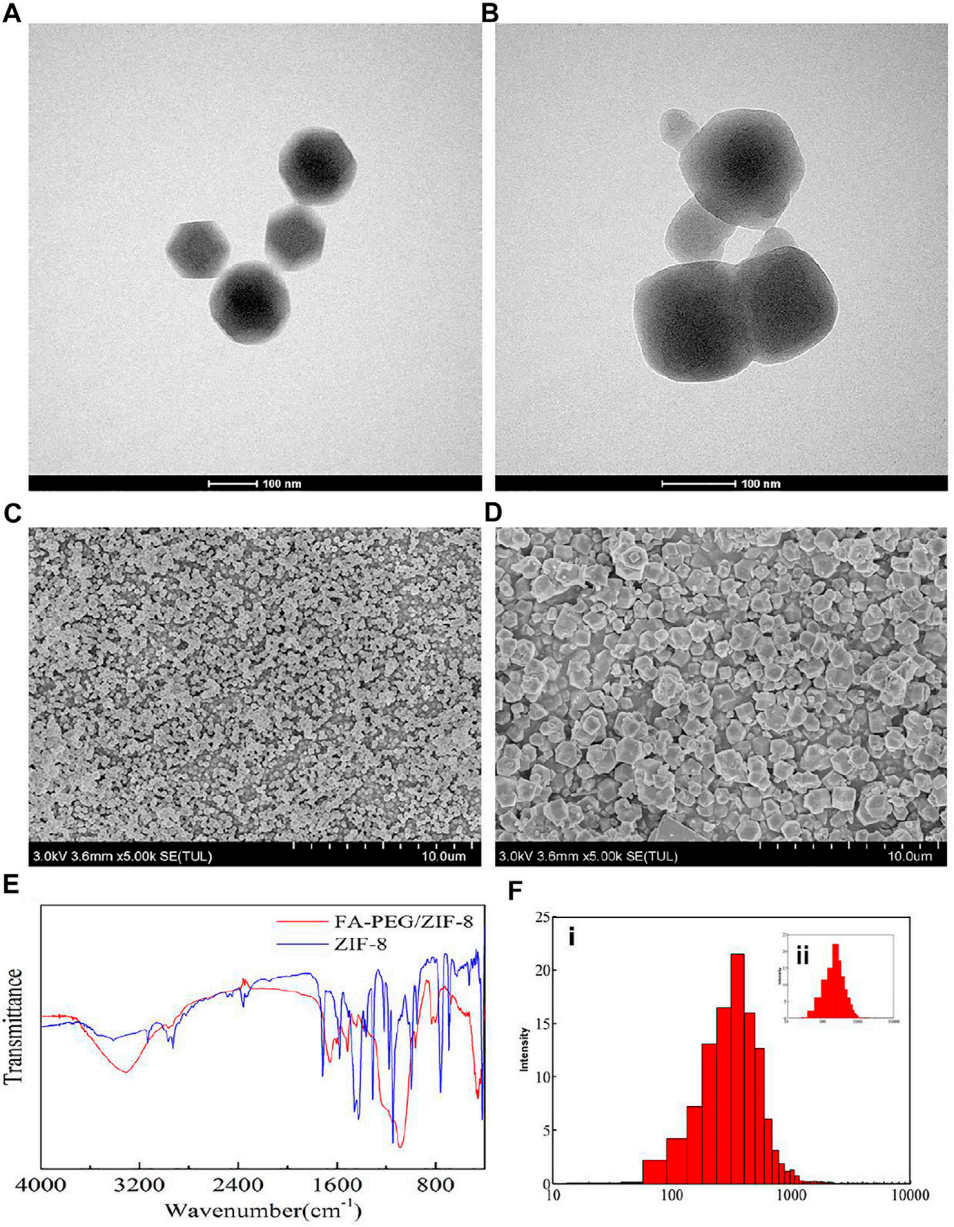
Characterization of ZIF-8 and FA-PEG/ZIF-8. TEM and SEM images of ZIF-8 **(A,C)** and FA-PEG/ZIF-8 **(B,D)**. FTIR spectra **(E)** and DLS **(F-i)** of FA-PEG/ZIF-8, DLS **(F-ii)** of ZIF-8.

The FA-PEG/ZIF-8 was synthesized by adding FA-PEG to a Zn(NO_3_)_2_·6H_2_O (0.66 mM) and 2-methylimidazole mixture (dispersed in methanol) in a dropwise manner. The morphology of ZIF-8 and FA-PEG/ZIF-8 was characterized by SEM and TEM, showing that the isolated nanoparticles ≈180 nm had a ball-like shape (Figures 1A–D). We further analyzed the FTIR spectra and XRD of ZIF-8 and FA-PEG/ZIF-8, indicating that FA-PEG had been successfully loaded into the ZIF-8 (Figure 1E and Supplementary Figure S1). Moreover, the DLS results showed the homogeneous distribution of ZIF-8 and FA-PEG/ZIF-8 (Figure 1F). These results suggested that FA-PEG/ZIF-8 was an ideal platform.

### Administration of FA-PEG/ZIF-8 to LPS-induced M/Ms at proper concentrations induce ROS generation and apoptosis

**FIGURE 2:**
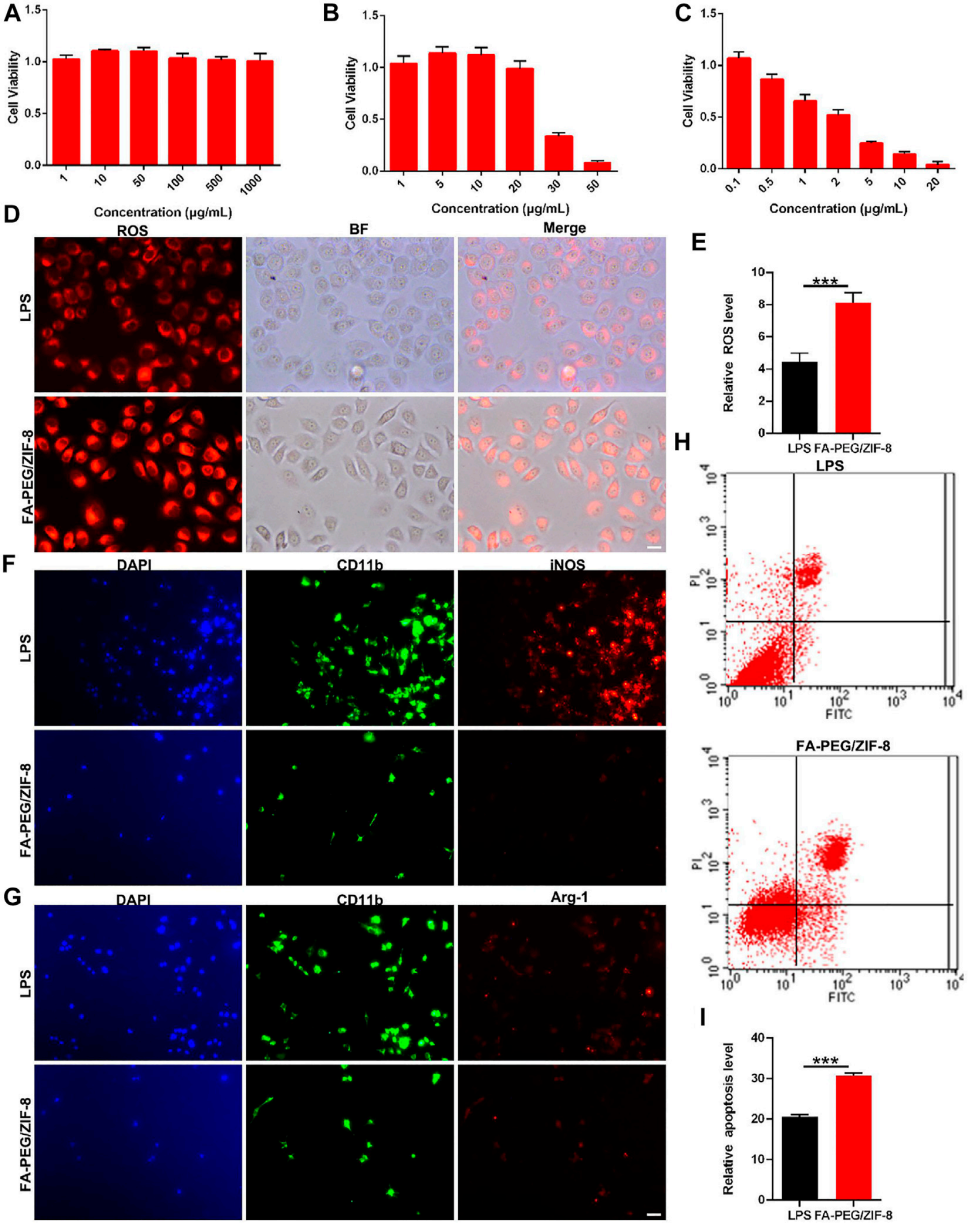
The cell viability and anti-inflammation effect of FA-PEG/ZIF-8 *in vitro*. Quantification analysis of cell viability in RAW264.7 cells treated with ZIF-8 **(A)**, FA-PEG **(B)**, and FA-PEG/ZIF-8 **(C)**. Representative images and **(D)** quantification analysis **(E)** of ROS activity in RAW264.7 cells. Scale bars = 50 µm. Representative images of iNOS **(F)** and Arg-1 **(G)** in RAW264.7 cells *via* immunofluorescence analysis. Scale bar = 50 μm. Representative images **(H)** and semiquantitative analysis **(I)**. Scale bars, 10 µm. Data presented as the mean±SD. Two-tailed Student’s t-test. ***p < 0.001.

The viability of RAW264.7 cells was investigated in the absence and presence of FA-PEG, ZIF-8, and FA-PEG/ZIF-8 based on MTT experiments (Figures 2A–C). After RAW264.7 cells were treated with LPS (1 μg/mL) for 24 h, the cell viability was near 100% with FA-PEG at various concentrations from 0–1000 μg/mL, and the cell viability was near 40% when the concentrations of 30 μg/mL ZIF-8 and the cell viability was near 50% at the concentration of 2 μg/mL FA-PEG/ZIF-8. Based on the cell viability results, FA-PEG/ZIF-8 treated up to 2 μg/mL were chosen for the LPS-induced activated RAW264.7 cells *in vitro.*

Oxidative stress is a basic protection mechanism and participates in the regulation of life activities, such as cell signal transduction, cell proliferation, and apoptosis (Jia et al., 2012; Anjum et al., 2020). However, under pathophysiological conditions (such as SCI), excessive free radicals will initiate inflammation and cell death in the acute phase (Yip and Malaspina, 2012; Alkabie and Boileau, 2016). We further analyzed the cellular generation of ROS. As shown in Figure 2D, we indicated that massive RAW264.7 cells were positive in the LPS group by ROS immunostaining, and were greatly induced in the FA-PEG/ZIF-8-treated groups. The quantification of the fluorescence level revealed that the administration of FA-PEG/ZIF-8 increased the ROS level in LPS-induced cells under inflammation conditions (Figure 2E).

M/Ms mediated the inflammatory, ROS, and apoptosis progress by the transformation of the polarization state in the SCI process. The activation pathway of macrophages mainly includes classical activation pathways (proinflammatory phenotype, iNOS, and IL-1β) and alternative activation pathways (anti-inflammatory phenotype, Arg-1, and IL-10) (Italiani and Boraschi, 2014; Landis et al., 2018; Zhou et al., 2020). The results showed that the level of iNOS^+^ cells was significantly decreased after treatment with FA-PEG/ZIF-8 (Figure 2F). Meanwhile, the level of Arg1^+^ cells was indifferent in the FA-PEG/ZIF-8 group as compared to that of the LPS group (Figure 2G). In summary, these results showed that the expression of iNOS has significantly declined in LPS-induced RAW264.7 cells following treatment with FA-PEG/ZIF-8, accompanied by the significant reduction of toxic RAW264.7 cells, indicating that FA-PEG/ZIF-8 promoted proinflammatory M/Ms death. We further extended the apoptosis levels of cells treated with or without FA-PEG/ZIF-8. The apoptosis percentage of LPS-induced proinflammatory RAW264.7 cells increased to about 20.66% (Figure 2H). The percentage of apoptosis proinflammatory RAW264.7 cells significantly increased when cells were incubated with LPS and FA-PEG/ZIF-8 (Figure 2I).

Based on these results, we demonstrated the targeting and anti-inflammation effects of FA-PEG/ZIF-8 on proinflammatory M/Ms *in vitro*.

### Application of FA-PEG/ZIF-8 to SCI mice at proper concentration promotes functional recovery and histological improvement

**FIGURE 3:**
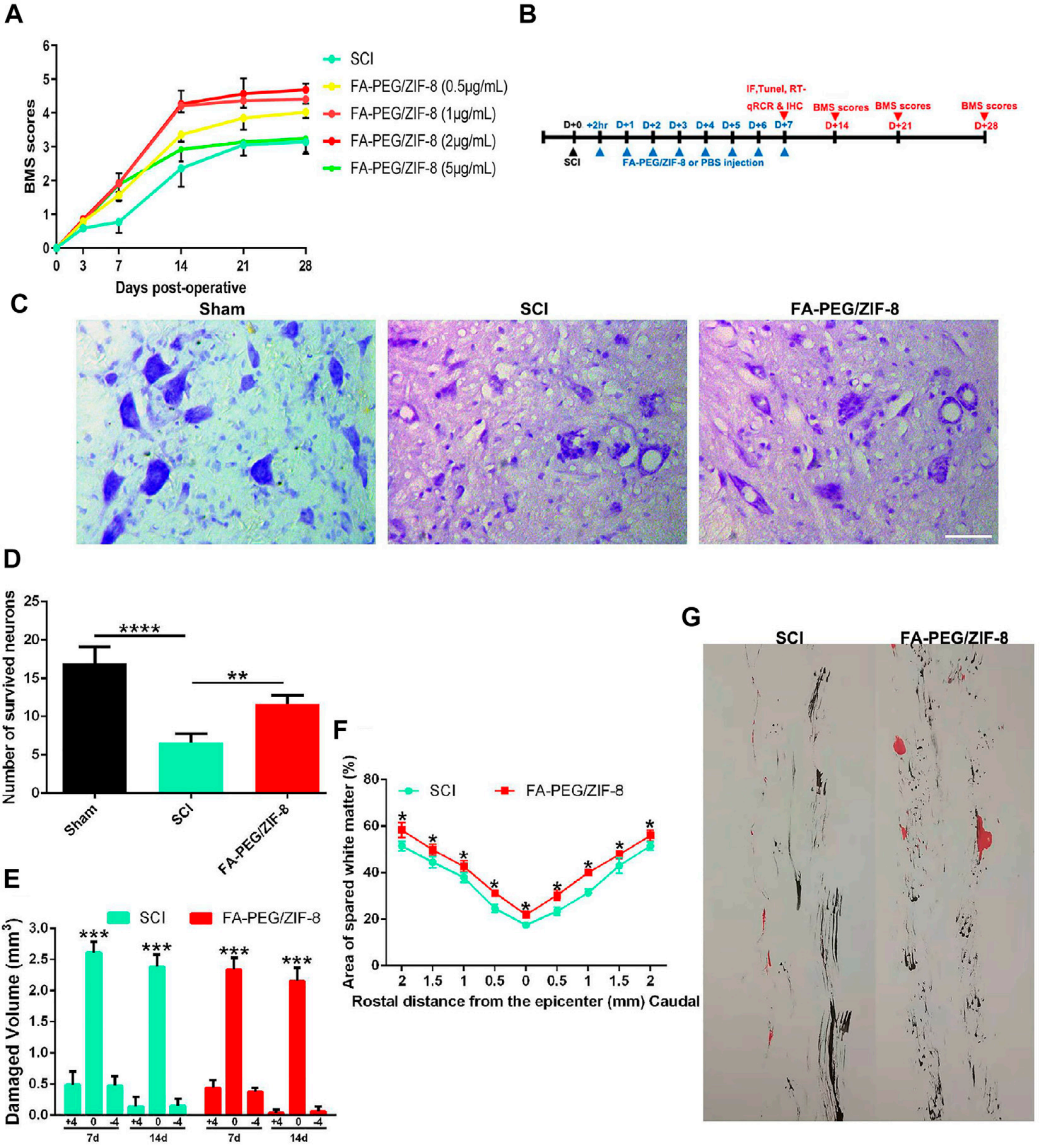
Therapeutic efficacy of FA-PEG/ZIF-8 in SCI mice. Quantification analysis of BMS scores in the SCI mice treated with various concentrations of FA-PEG/ZIF-8. **(A)** Two-way ANOVA (repeated measures) with Bonferroni’s post-tests. The experimental time of this study. **(B)** Representative images **(C)** and quantification analysis **(D)** of neurons in the spinal cord. Quantification analysis of damaged volume **(E)** in SCI and FA-PEG/ZIF-8 groups 7 and 14 days post-injury. Quantification analysis of the lesion area percentage **(F)** in mice. Two-way ANOVA (repeated measures) with Bonferroni’s post-tests.Representative images of the giant experiment **(G)** in SCI and FA-PEG/ZIF-8 groups in 28 days post-injury. Scale bars, 100 µm. Data are presented as the mean±SD. Two-tailed Student’s t-test.*p < 0.1, **p < 0.01, ***p < 0.001, ****p < 0.0001.

To further investigate the targeting and effects of FA-PEG/ZIF-8 in *in vivo* responses, we used a moderate spinal cord contusion at the T10 level in the mice models, and the FA-PEG/ZIF-8 were injected once at these concentrations: 0.5, 1, 2, and 5 μg/mL. As shown in Figures 3A,B, the proper concentration (2 μg/mL≈10 μg/Kg) of FA-PEG/ZIF-8 was a benefit to injured mice for a longer period of four weeks. Moreover, the neurons were lost in the SCI group, and the group treated with FA-PEG/ZIF-8 posed a highly substantial improvement in the survival neurons of the ventral horn in post-injury 4 weeks (Figures 3C,D).

After SCI on days 7 and 14 following the treatment of FA-PEG/ZIF-8, the cavity size decreased significantly compared to that of the SCI group (Figure 3E). Additionally, the staining result revealed that decreased area of spared white matter in the lesion cavity after SCI was remarkably increased following the treatment of FA-PEG/ZIF-8 (Figure 3F). The images of the mice’s gait experiment revealed a substantial function recovery improvement after the treatment of FA-PEG/ZIF-8 (Figure 3G).

Taken together, the proper concentrations of FA-PEG/ZIF-8 (2 μg/mL≈10 μg/Kg) were beneficial to the SCI mice for the long-term *in vivo* experiment.

### Treatment of FA-PEG/ZIF-8 to the expression of nerve cells and immune cells in uninjured mice

**FIGURE 4:**
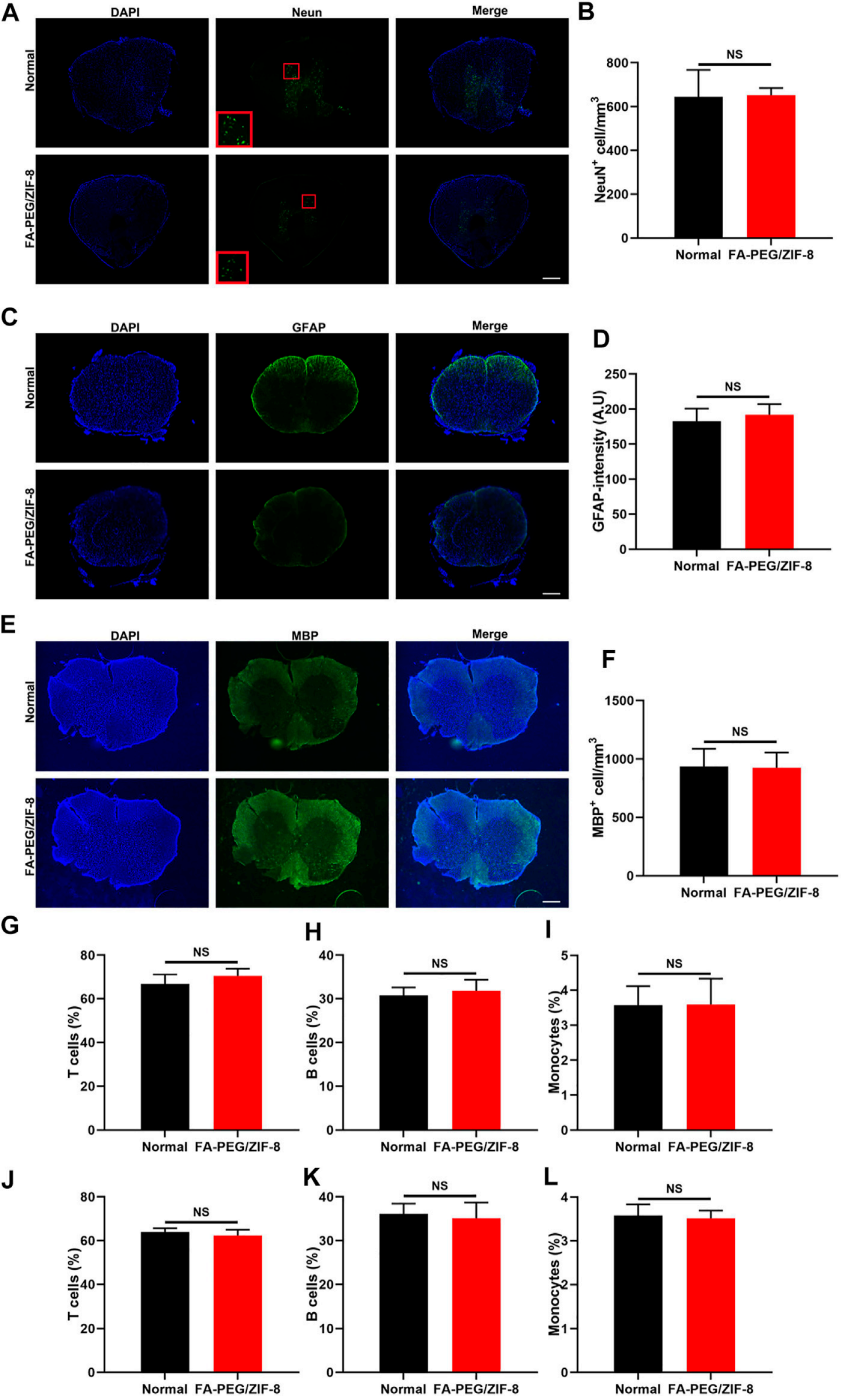
The targeting effect of FA-PEG/ZIF-8 *in vivo*. Representative expression and quantification analysis of Neun **(A,B)**, GFAP **(C,D)**, and MBP **(E,F)** in FA-PEG/ZIF-8 and WT groups. Scale bars = 200 µm. Quantification analysis of T cells **(G,J)**, B cells **(H,K)**, and monocytes **(I,L)** in the blood and spleen of FA-PEG/ZIF-8 and WT groups. Data are presented as the mean±SD. Two-tailed Student’s t-test.

To assess whether FA-PEG/ZIF-8 specifically targets M/Ms without affecting other major cell types in the spinal cord, we investigated the cell-to-cell differences. As shown in Figures 4A–F, we found no difference between the two groups in the number of neurons and oligodendrocytes and the fluorescence intensity of astrocytes.

In addition, there was no indifference in leukocyte populations in the blood and spleen after being treated with FA-PEG/ZIF-8 compared to normal control mice (Figures 4G–L). These data indicated that FA-PEG/ZIF-8 treatment was an effective therapy to target, having few side effects on neural and glial cells and that did not influence the population of peripheral immune cells to disturb the circulating immunity.

### Administration of FA-PEG/ZIF-8 to the reduction of proinflammatory M/Ms and inflammatory cytokines in the acute phase of SCI

**FIGURE 5:**
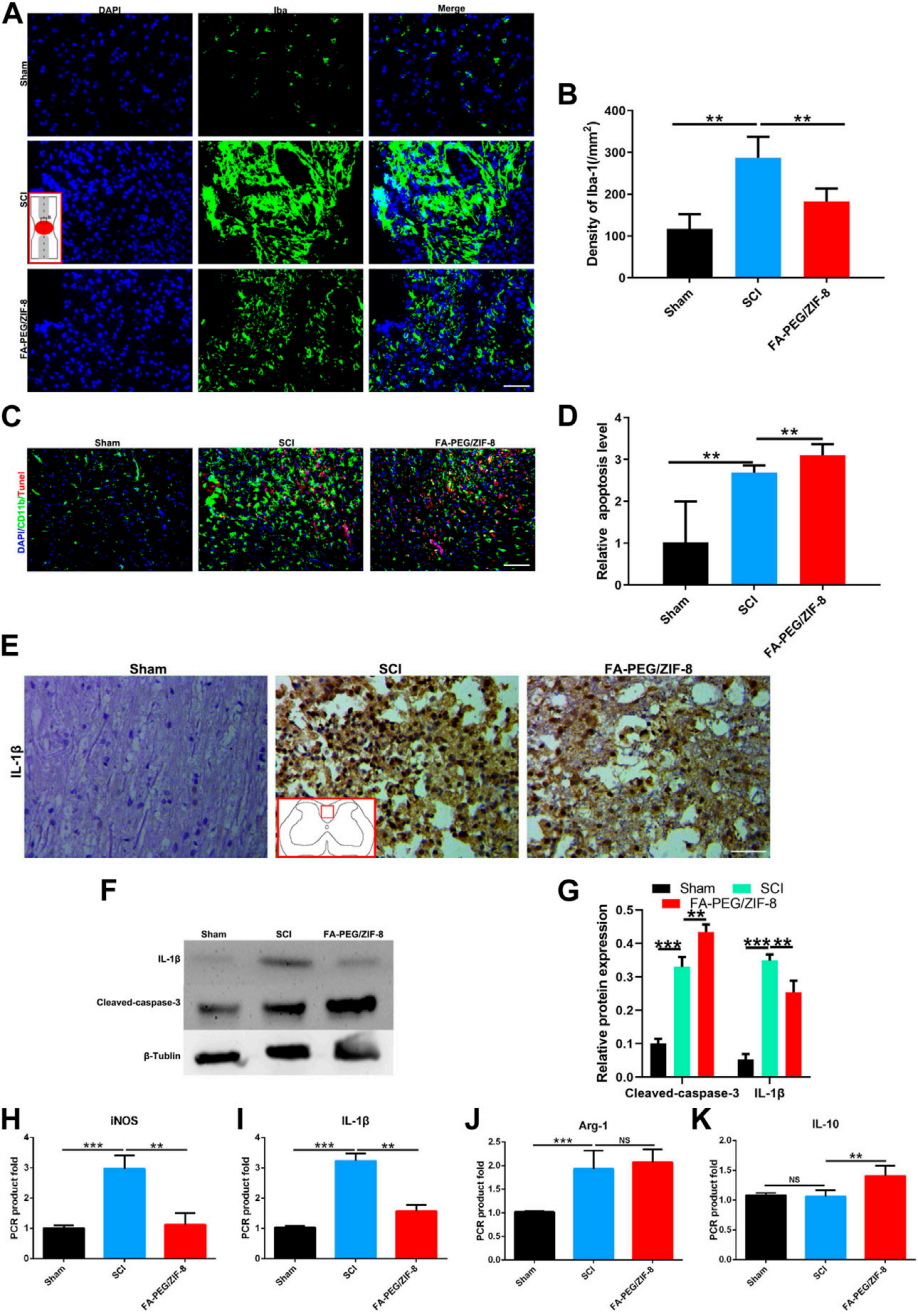
Anti-inflammation effect of FA-PEG/ZIF-8 in SCI mice. Representative images **(A)** and quantification analysis **(B)** of Iba-1 in the spinal cord. Representative images **(C)** and quantification analysis **(D)** of Tunel in the spinal cord. Representative images **(E)** of IL-1β in the spinal cord. Representative images **(F)** and quantification analysis **(G)** of IL-1β and cleaved-caspase-3 in the spinal cord *via* Western blot. Quantification analysis of iNOS **(H)**, IL-1β **(I)**, Arg-1 **(J)**, and IL-10 **(K)** mRNA levels in the spinal cord. Scale bars, 100 µm. Data are presented as the mean±SD. Two-tailed Student’s t-test.**p < 0.01, ***p < 0.001, ****p < 0.0001.

To further study the relationship between the underlying molecular mechanism and function recovery in the acute phase (7 days post-injury), we tested the M/Ms level in the injured focal by immunofluorescence staining for Iba-1. At 7 days of SCI, massive cells were stained positive for Iba in the SCI group, and the stained cells decreased in the group treated with FA-PEG/ZIF-8 (Figures 5A,B). The increase of apoptosis expression was tested by CD11b and Tunel double-positive staining (Figures 5C,D) and Western blot (Figures 5F,G) in the FA-PEG/ZIF-8 group, compared to sham and SCI groups. Moreover, we found that FA-PEG/ZIF-8 treatment significantly decreased the percentage of positive iNOS in Iba-1 positive cells and increased the percentage of positive Arg-1 in Iba-1 positive cells (Supplementary Figure S2). These results showed that FA-PEG/ZIF-8 administration significantly decreased proinflammatory M/Ms in the injured spinal cord.

A decrease in IL-1β expression was found in the FA-PEG/ZIF-8 group as compared to those of sham and SCI groups *via* immunohistochemical staining (Figure 5E). Furthermore, in the FA-PEG/ZIF-8 group, the reduction of iNOS (Figure 5H) and IL-1β (Figure 5I) mRNA expressions were presented similar to previous results *in vitro* (Figure 2F), the level of Arg-1 had no changes (Figure 5J), and the mRNA expression of IL-10 (Figure 5K) significantly increased as compared to the SCI group. Thus, these data confirmed that FA-PEG/ZIF-8 suppressed proinflammatory M/Ms and subsequently downregulated proinflammatory cytokines. Compared with the various previously reported drugs, FA-PEG/ZIF-8 had a better therapeutic effect on SCI rats because the damage recovered faster (Table 2).

**TABLE 2:** Comparison of the motor function recovery after SCI by using different medicines.

Nanomedicines	Motor function recovery	Therapeutic effects	Advantages/Disadvantages	Ref
Glutathione	Improves after 2 weeks	Antioxidant	Reduces inflammation; *very low bioavailability*	del Rayo Garrido et al. (2013)
Methylprednisolone	Improves after 15 days	Immunosuppressant	Reduces inflammation; *water-sodium retention, susceptible to infection*	Barth et al. (2004)
CeO_2_	Improves *in vitro* biocompatibility and auto-recovery abilities	Delivered a bone regeneration drug	Increases biocompatibility of the drug; *not available for injection and the in vivo effects are unknown*	Dong et al. (2020)
CQDs	Improves significantly after 8 weeks	Reduced inflammation, astrogliosis, and apoptosis induced by secondary injury	Remarkable protective effect for nerves; *not cost-effective, toxic concern*	Luo et al. (2020)
FA-PEG/ZIF-8	Improves significantly after 7 days	Inflammation-suppressing	Reduces inflammation, injectable, low toxicity, cost-effective, simple preparation	Current work

Note: CQDs, carbon quantum dots.

### Biological safety of FA-PEG/ZIF-8

**FIGURE 6:**
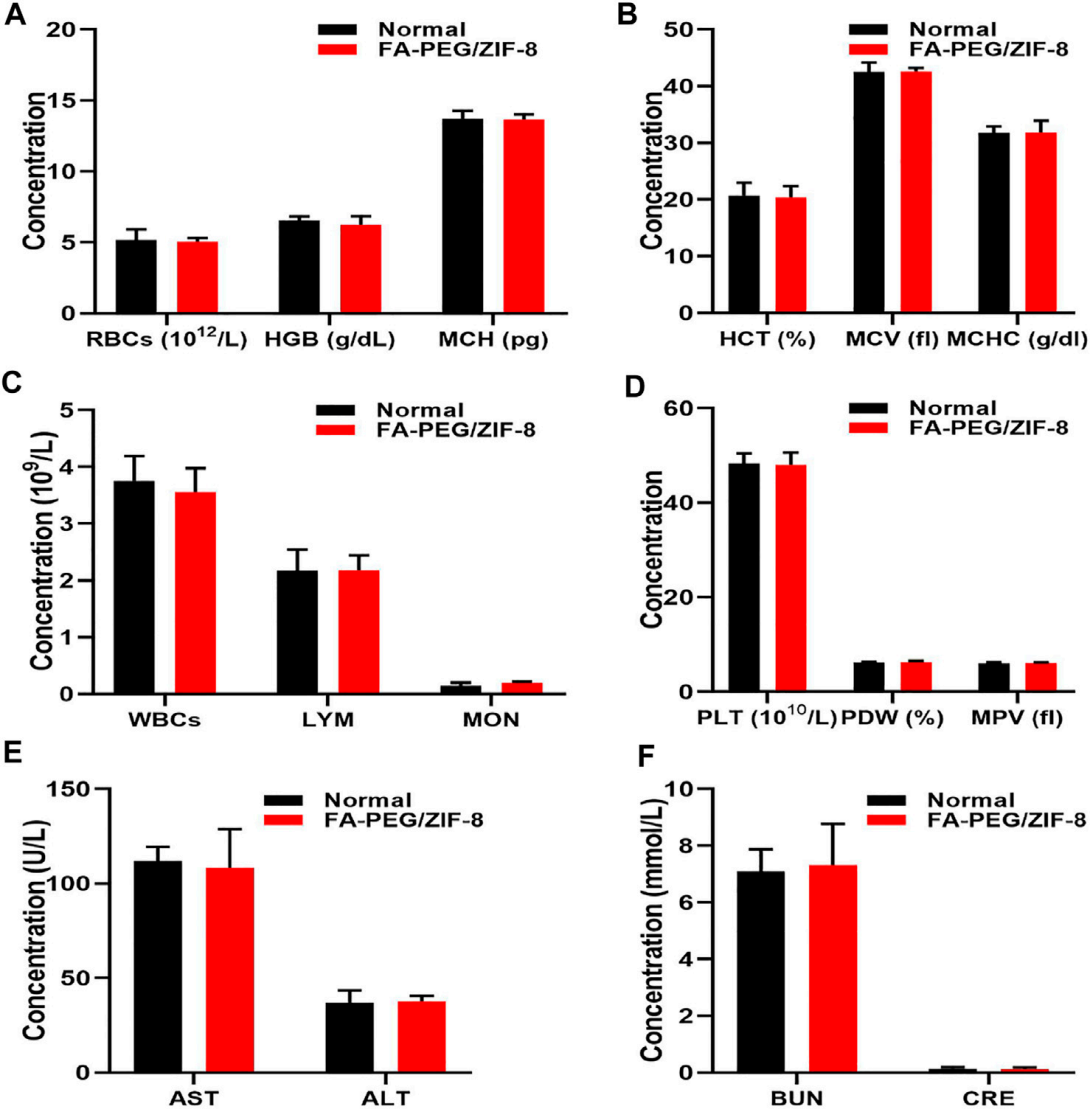
*In vivo* biocompatibility assessment of FA-PEG/ZIF-8. Blood parameters **(A–D)** in normal and FA-PEG/ZIF-8 groups. Quantification analysis of AST, ALT **(E)**, BUN, and CRE **(F)**. Data are presented as the mean±SD. Two-tailed Student’s t-test.

Furthermore, we evaluated the biocompatibility of FA-PEG/ZIF-8 (100 μg/kg, 10-fold higher than the concentration used to treat SCI mice) for blood chemistry. Blood analysis data (Figures 6A–D) showed no difference between the FA-PEG/ZIF-8 group and normal mice. Serum biochemistry (Figures 6E,F) showed aspartate aminotransferase (AST), alanine aminotransferase (ALT), blood urea nitrogen (BUN), and creatinine (CRE) in mice treated with FA-PEG/ZIF-8. Good biocompatibility with the normal group, liver, and kidney. Collectively, these results suggest that FA-PEG/ZIF-8 has little *in vivo* toxicity.

## Discussion

Neuroinflammation has been implicated in the pathophysiology of CNS diseases, such as Alzheimer’s disease (Eikelenboom et al., 2010), SCI (Pang et al., 2021), and aging (Zia et al., 2021). Targeting M/Ms-mediated immune responses are promising in the treatment of these diseases (Stoll and Jander, 1999; Minogue, 2017). Due to the excellent biodegradability and bioactivity, nanomaterials have been reported to inhibit inflammation and promote recovery (Wu et al., 2019). In our study, we fabricated FA-PEG/ZIF-8 nanoparticles and found that 1) FA-PEG/ZIF-8 inhibited LPS-induced inflammatory responses by targeting M/Ms; 2) SCI-induced M/Ms activation is superior to its subsequent neuronal loss; and 3) FA-PEG/ZIF-8-mediated treatment efficiency has a large practical impact on SCI recovery.

M/Ms are typically activated after injury and secrete proinflammatory cytokines such as TNF-α and IL-1β. However, another phenotype of M/Ms (expressing Arg-1, IL-10) may have less impact in the early stages of SCI compared with proinflammatory M/Ms (Fan et al., 2018). This is not surprising, given that proinflammatory M/Ms may be more potent than other nerve cells that contribute to neuroinflammation. Recently, nanomaterials with inflammation-regulatory capacities have been studied to broaden therapeutic strategies for many diseases (Padmanabhan and Kyriakides, 2015). Compared to traditional drugs, nanotherapeutics exhibit multiple advantages such as reduced dosing frequency, decreased adverse events, and enhanced accumulation in diseased tissues. Consequently, various nanoformulations, such as liposomes (Wang et al., 2021), fibers (Xi et al., 2020), and metal nanoparticles (Lin et al., 2021), have been extensively developed for anti-SCI treatments. However, utilization of the microenvironment of SCI to “smartly” target M/Ms and the mechanism of nanotherapies for targeted treatment of M/Ms still need to be further investigated. Therefore, we synthesized FA-PEG-modified ZIF-8 nanoparticles (FA-PEG/ZIF-8) with M/Ms-targeting ability.

In response to LPS-induced M/Ms activation, we found that FA-PEG/ZIF-8 markedly improved the expression of Arg-1 and inhibited the expression of iNOS *in vitro*. Excitingly, the apoptosis of proinflammatory M/Ms induced by FA-PEG/ZIF-8 treatment was increased. In the combination of M/Ms polarization and proinflammatory M/Ms apoptosis, we observed the increase of survival neurons and functional recovery in the FA-PEG/ZIF-8 group, as compared to SCI mice. These results verified that the selective regulation of proinflammatory M/Ms *in vivo* and *in vitro* treated by the FA-PEG/ZIF-8 administration promoted neuroprotective M/Ms polarization and improved SCI recovery. Another important finding from this study is the biosafety of FA-PEG/ZIF-8 in the SCI treatment. We used the complete blood panel analysis and serum biochemistry test as indexes for biosafety and found that FA-PEG/ZIF-8 had good biocompatibility. Moreover, compared with the various previously reported drugs, FA-PEG/ZIF-8 had a better therapeutic effect on SCI rats because the damage was fixed faster (Table 1).

In summary, these findings contribute to the anti-inflammatory effect of FA-PEG/ZIF-8 in the long-term maintenance of neuroprotective M/Ms in the injured spinal cord, providing a new specialized treatment for SCI.

## Conclusion

In this study, our results demonstrate that FA-PEG/ZIF-8 treatment reduces proinflammatory M/Ms in the injured spinal cord and induces the survival of ventral anterior horn neurons after SCI, thereby improving SCI motility function. In addition, we also found that the administration of FA-PEG/ZIF-8 significantly reduced the proinflammatory M/Ms ratio, inhibited neuroinflammation, and promoted functional recovery. Collectively, these results suggest that proinflammatory M/Ms may play a major critical role in the early development of SCI.

## Data Availability

The original contributions presented in the study are included in the article/Supplementary Material. Further inquiries can be directed to the corresponding authors.

## Abbreviations

CNS, central nervous system; FR, folate receptors; FA, folic acid; M/Ms, microglia/macrophages; MOFs, metal–organic frameworks; PEG, polyethylene glycol; PLTs, platelets; RBCs, red blood cells; ROC, receiver-operating characteristics; ROS, reactive oxygen species; SCI, spinal cord injury; WBCs, white blood cells; ZIF-8, zeolitic imidazolate frameworks.
