# Corrigendum: Therapy of spinal cord injury by folic acid polyethylene glycol amine-modified zeolitic imidazole framework-8 nanoparticles targeted activated M/Ms

**DOI:** 10.3389/fbioe.2023.1200532

**Published:** 2023-05-04

**Authors:** Qi Li, Yue Guo, Chang Xu, Jiachen Sun, Fanzhuo Zeng, Sen Lin, Yajiang Yuan

**Affiliations:** ^1^ Department of Orthopedics, First Affiliated Hospital of Jinzhou Medical University, Jinzhou, China; ^2^ Key Laboratory of Medical Tissue Engineering, Jinzhou Medical University, Jinzhou, China

**Keywords:** spinal cord injury, microglia/macrophages, metal-organic frameworks, inflammation, apoptosis same as

In the published article, there was an error in Figure 5C as published the authors noticed one error image (SCI group) in Figure 5C. The corrected Figure 5C can be appear below.

**FIGURE 5:**
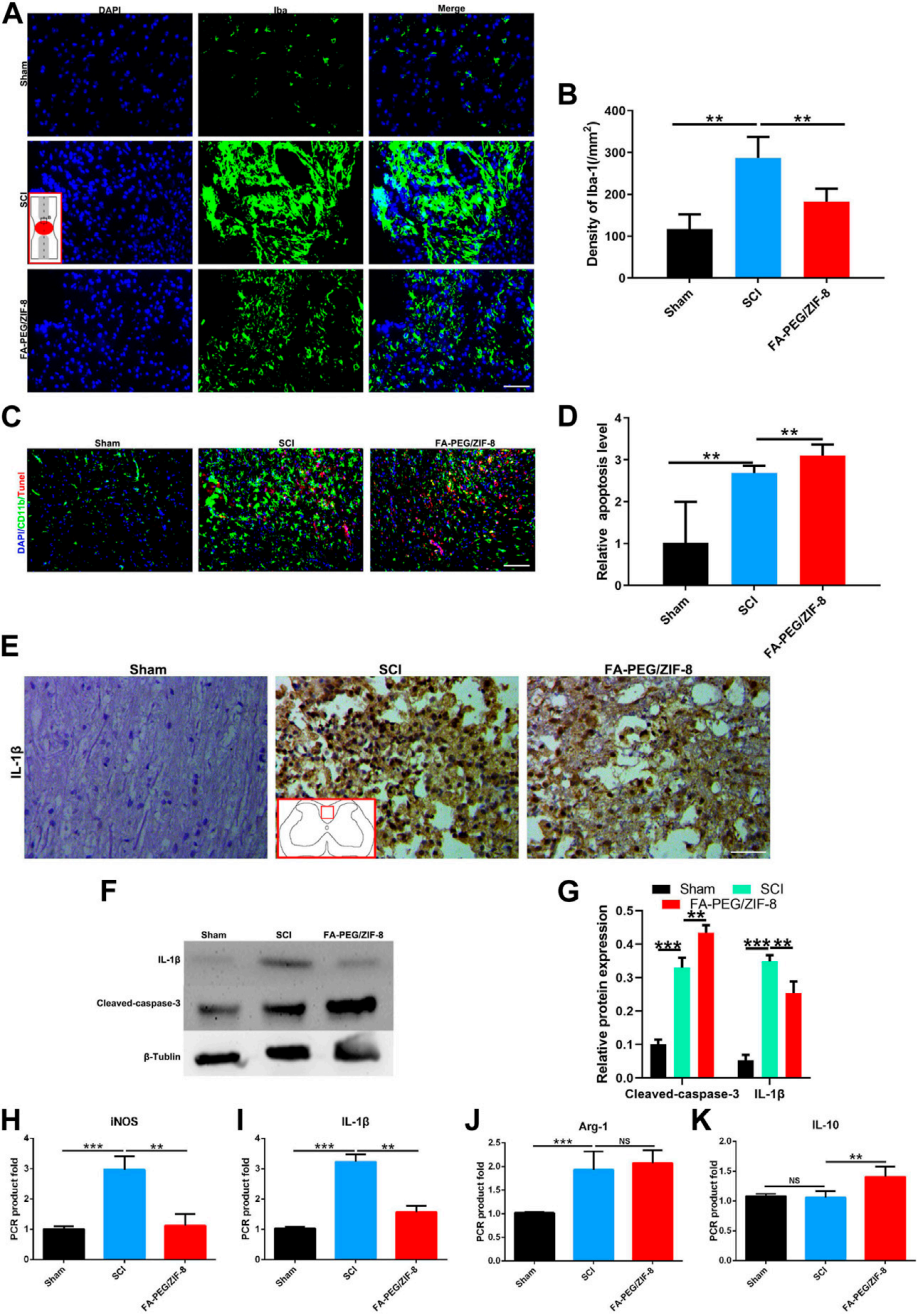
Anti-inflammation effect of FA-PEG/ZIF-8 in SCI mice. Representative images **(A)** and quantification analysis **(B)** of Iba-1 in the spinal cord. Representative images **(C)** and quantification analysis **(D)** of Tunel in the spinal cord. Representative images **(E)** of IL-1β in the spinal cord. Representative images **(F)** and quantification analysis **(G)** of IL-1β and cleaved-caspase-3 in the spinal cord *via* Western blot. Quantification analysis of iNOS **(H)**, IL-1β **(I)**, Arg-1 **(J)**, and IL-10 **(K)** mRNA levels in the spinal cord. Scale bars, 100 µm. Data are presented as the mean±SD. Two-tailed Student’s t-test.**p < 0.01, ***p < 0.001, ****p < 0.0001.

The authors apologize for this error and state that this does not change the scientific conclusions of the article in any way. The original article has been updated.

