# Supplementary material for: Therapy of spinal cord injury by folic acid polyethylene glycol amine-modified zeolitic imidazole framework-8 nanoparticles targeted activated M/Ms
Source: Front Bioeng Biotechnol. 2022 Sep 15;10:959324. doi: 10.3389/fbioe.2022.959324 (PMC9519986; doi:10.3389/fbioe.2022.959324)
Supplement: Supplementary file 1 [file DataSheet1.PDF]

Supplement materials

Therapy of spinal cord injury by folic acid polyethylene glycol amine-modified zeolitic imidazole framework-8 nanoparticles targeted activated M/Ms

Qi Li[1,#], Yue Guo[2,#], Chang Xu[1,#], Jiachen Sun[1], Fanzhuo Zeng[1], Sen Lin[2,*], Yajiang Yuan[1,2,*]

[1] Department of Orthopedics, First Affiliated Hospital of Jinzhou Medical University, Jinzhou, P. R. China.

[2] Key Laboratory of medical tissue engineering, Jinzhou Medical University, Jinzhou, P. R. China.

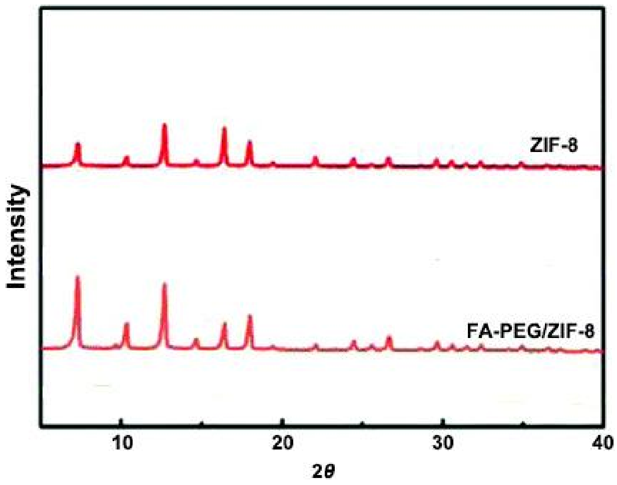

Figure S1. XRD of ZIF-8 and FA-PEG/ZIF-8.

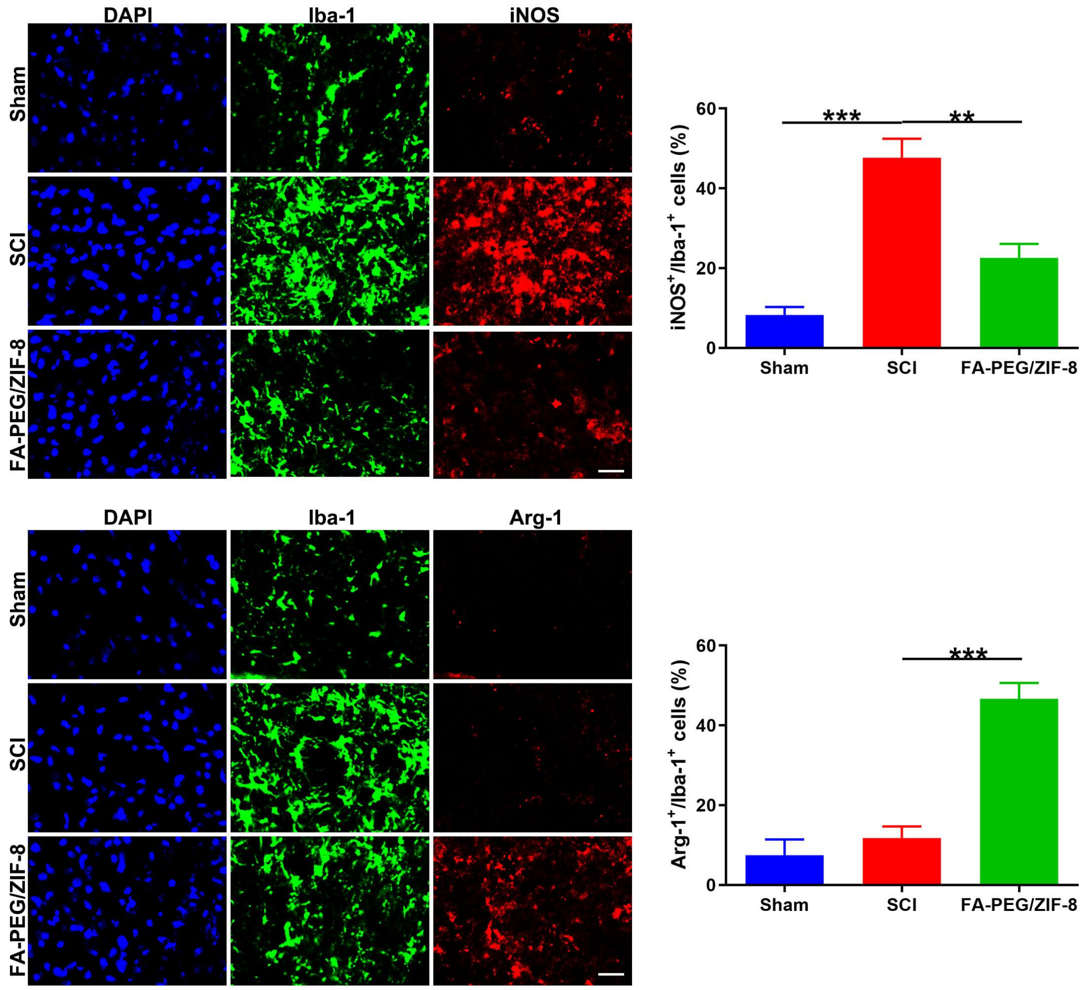

Figure S2. FA-PEG/ZIF-8 improved injured spinal cord on days 7 after SCI. Scale bars, 100 μm. Data were mean ± SD. Two-tailed Student's t-test.
